# A whole system approach to promoting health and human performance in military settings as *vital* prerequisites for force readiness and operational capability

**DOI:** 10.3389/fphys.2025.1541256

**Published:** 2025-04-08

**Authors:** Joanne L. Fallowfield, Jace R. Drain, Julia Carins, Helen Kilding, Emma Williams, Ben Fisher, Debra Hayhurst, Alysia Gourlay, Simon Olivotto, Garrett Bullock

**Affiliations:** ^1^ Institute of Naval Medicine, Ministry of Defence, Alverstoke, Hampshire, United Kingdom; ^2^ Joint Health Command, Department of Defence, Canberra, ACT, Australia; ^3^ Social Marketing @ Griffith, Griffith Business School, Griffith University, Nathan, QLD, Australia; ^4^ Sports Performance Research Institute New Zealand, Auckland University of Technology, Auckland, New Zealand; ^5^ Defence Primary Healthcare, Headquarters Defence Medical Services, DMS Whittington Barracks, Lichfield, Staffordshire, United Kingdom; ^6^ Healthcare Governance Lead, Defence Medical Rehabilitation Centre Stanford Hall, Loughborough, Leicestershire, United Kingdom; ^7^ Department of Orthopaedic Surgery and Rehabilitation, Wake Forest University School of Medicine, Winston-Salem, NC, United States

**Keywords:** military, force readiness, health promotion, workplace intervention, whole system approach

## Abstract

The military role and associated occupation-specific training contribute to a high musculoskeletal injury (MSKI) incidence and poor health burden. A fit Force is better prepared for achieving mission success, as well as being more resilient to operational physical and cognitive demands. Conversely, MSKI and ill-health reduce Force readiness. Internationally, militaries have common workforce capacity and capability challenges, where more is being asked of fewer personnel. Unhealthy body composition, low aerobic fitness, poor movement control and poor health behaviours interact to adversely impact human performance. The military workplace—including leadership prioritisation and resource allocation—has generally *not* strategically managed and supported health and performance interventions to maximise people outcomes. Efforts have focused on the individual and *their* capabilities to address *their* ill-health or poor performance. Only through system-based thinking—adopting a Whole System Approach (WSA)—can effective evidence-based interventions to promote health and human performance be: holistically developed; successfully implemented at scale across geographically dispersed organisations to realise meaningful and enduring outcomes; and impacts measured and evaluated. This paper provides a synthesis of scientific and practice-based evidence to operationalise system-thinking in developing integrated WSA workplace interventions for military health and human performance, and measure effect and return on investment. Whilst militaries are recognising the need for a paradigm shift to realise the benefits of effective health and performance interventions, persuasive financial arguments could assist with overcoming large-organisation inertia. Moreover, system-based thinking—addressing individual and organisational factors—could maximise military health and performance, foster resilience and deliver operational effectiveness.

## 1 Introduction

Increasing global geopolitical unrest—manifesting as virtual and actual state-on-state or insurgency-on-state offensive actions—has increased nations’ requirements for a *healthy* and *ready* military. Good health is defined as “*complete physical, mental and social wellbeing… not merely the absence of disease*” (World Health Organization, 2020). Good military health, therefore, combines “general health” and role-related physical and cognitive fitness, ensuring readiness to deploy when required. Indeed, military readiness has been defined as “*The ability to deploy personnel and equipment within a prescribed timeframe, for personnel to be trained to effectively use that equipment and for deployments to be sustained until all mission objectives are accomplished*” (House of Commons Defence Committee, 2024). Thus, military-specific health and fitness should provide the foundation to any human performance, enhancement and/or augmentation technology.

However, persistent ill-health in military populations—including musculoskeletal injury (MSKI)—arising from occupational demands and training required to meet these demands (Wardle and Greeves, 2017; Hauschild et al., 2017; de la Motte et al., 2017), impacts readiness. A fit military is better able to achieve mission success, as well as ensuring increased robustness to withstand arduous operations (Nindl et al., 2015; Szivak and Kraemer, 2015). Conversely, poor health and MSKI impact readiness to deploy and operational effectiveness due to loss of qualified personnel (e.g., temporary or permanent medical restrictions), and lost duty time. Internationally, militaries have common workforce challenges—under-recruitment of new trainees and continued workforce outflows are reducing Armed Forces’ size (Commonwealth of Australia, 2024; UK Parliament, 2023; United States Government Accountability Office, 2023)—which means more is asked of fewer personnel. Moreover, personnel are increasingly required to fulfil more specialist technical roles across five modern warfare domains (land, maritime, air, space and cyberspace). Every soldier, sailor and aviator is required to be a *Force multiplier*; more present, more capable and more resilient.

To meet these challenges, militaries are developing health and human performance programmes. Human performance *“enhancement and augmentation”* refers to practices and technologies that *extend* physical, physiological or cognitive performance beyond normal human limits (Adlakha-Hutcheon et al., 2021). Nevertheless, without the necessary prerequisites of health and fitness, the benefits of enhancement and augmentation interventions will be, at best, suboptimal. At worst, they have direct (e.g., impacts on health, physical and/or cognitive capabilities) and indirect (e.g., undermine organisational and/or individual responsibilities for health and fitness) adverse consequences. Thus, the start-point for enhancing readiness and operational capability in military personnel must be ensuring good health and role-related fitness.

Health and performance initiatives within military organisations have failed to realise enduring outcomes (Fallowfield and Carins, 2025). Such initiatives have generally been individual-focussed, whilst not addressing systemic (environment and social) factors, which prevent individuals enacting the desired behaviours, as well as the challenges of implementing population-level complex interventions (Fallowfield and Carins, 2025). Military ill-health impacts readiness, degrading the ability to generate and sustain an effective people *capability* and reduces Force *capacity* (Molloy et al., 2020).

This paper provides a synthesis of scientific and applied practice-based evidence to present a framework for developing a context-specific, integrated Whole System Approach (WSA) for military health and performance, supporting readiness and capability imperatives. Exemplars of *published* initiatives and their limitations are discussed. We detail a methodology for operationalising a WSA in the military setting, before considering identification of system process (activity) and outcome metrics. There is a recognised need for a paradigm shift in organisational policies and behaviours to realise enduring positive health and performance outcomes (Teyhen et al., 2018); actionable strategies and persuasive financial arguments could assist overcome large-organisation inertia.

## 2 Established military human performance models

Increased professionalisation of sport has been associated with significant investment in human performance programmes and supporting technologies. Similar initiatives within military settings have also gained traction (Deuster and O'Connor, 2015); published examples include the U.S. Army’s Tactical Human Optimization Rapid Rehabilitation and Reconditioning (THOR3) and Holistic Health and Fitness (H2F) programmes (Grier et al., 2018; Whitehurst et al., 2024). The primary driver for human performance programmes is their potential benefit to Force readiness. Strong evidence supports their effectiveness in military populations, contributing to enhanced occupational performance and organisational capability (Burley et al., 2020; Knapik et al., 2012; Vaara et al., 2022; Grier et al., 2018). However, performance *per se* is not the primary impediment to the physical readiness of personnel; rather, MSKI represents the greatest risk (Molloy et al., 2020). THOR3 and H2F are considered *human performance* programmes, but they include significant medical (healthcare and rehabilitation) elements, to realise expedient treatment and improved patient outcomes compared with traditional military medical systems (Whitehurst et al., 2024; Grier et al., 2018).

Human performance programmes do have a role in MSKI mitigation (Rhon et al., 2022; Ladlow et al., 2022; Mooney et al., 2017). However, human performance systems and medical systems are often treated as separate entities (Teyhen et al., 2018; Tenan and Alejo, 2024), when they should be considered as integrated elements of a WSA promoting Force health protection, readiness and operational capability. To achieve a WSA to health and readiness it is suggested that both medical and human performance systems must adapt to better serve military organisations’ needs. Medical systems should place greater emphasis on preventative medicine (e.g., health surveillance and risk mitigation interventions) in addition to clinical service delivery, while human performance systems need to achieve greater alignment with health and medical systems’ outcomes (Tenan and Alejo, 2024). Arguably, many human performance activities are health (primary prevention) activities, given that MSKI mitigation is often a stated objective (Drew et al., 2023). Equally, many primary preventative activities initiated by medical practitioners could also be described as human performance activities. This overlap and interdependency are unquestionable; indeed, they should be considered as a unified *System for Health* (Teyhen et al., 2018).

Equally important to the health and performance of personnel is the “training and employment system” responsible for generating and maintaining the military workforce. *Ab initio* training continuums are important for developing foundational and trade-specific military skills and physical fitness, but are also associated with a high MSKI incidence (Molloy et al., 2020). Occupational training and task exposure—essential for role performance—are similarly associated with MSKI risk (Roy et al., 2012; Schuh-Renner et al., 2017), but is a modifiable risk (Sammito et al., 2021). Thus, training requirements and readiness need to be balanced against ill-health (MSKI) risk. Improved integration between human performance, medical, and military training and employment systems could maximise the health and performance that underpins readiness and operational capability. Through an elite sport lens, this has been described as an *integrated performance health management and coaching model* (Dijkstra et al., 2014). This would require significant changes in military culture and ways of working (Teyhen et al., 2018). Moreover, a health and performance WSA requires responsibility and accountability at *all* levels of the chain of command—new-entry trainees to senior leaders—to realise readiness and performance objectives.

## 3 Operationalising a whole system approach in the military setting

Effectively tackling complex, interconnected health problems—and realising enduring human performance outcomes—demands adoption of a WSA (Fallowfield and Carins, 2025). A WSA refers to integrated, multi-level, multi-component, multi-disciplinary interventions (Public Health England, 2019), which adopt a person-centred perspective that considers the motives and priorities driving individual behaviours (Fallowfield and Carins, 2025). Individuals are educated and empowered to develop health and performance improving capabilities, whilst recognising the influence of their context and opportunities for action, as described by the COM-B behaviour change model (Michie et al., 2011).

System-thinking has been applied to health behaviours in military workforces (Fallowfield and Carins, 2025), and conceptually elucidated specifically with respect to nutrition as a military capability (Fallowfield et al., 2024b). The purposeful application of system-thinking to health and performance more broadly is presently not part of mainstream military doctrine or practice. Realising the *readiness and capability dividend* from military health and human performance programmes will require organisations to adopt a WSA; setting organisation conditions that enable individuals to thrive.

Consistent with public health and MSKI mitigation models (Finch, 2006), a sequential framework supporting implementation and continuous improvement of a health and performance WSA in military settings is recommended. A six-step model has been proposed (Fallowfield et al., 2024c), comprising: Step-1, Set-up; Step-2, Consultation; Step-3, Planning; Step-4, Action; Step-5 Manage; and Step-6, Refresh and Reflect. This framework could be applied across a defined domain (e.g., squadron, company, unit), or organisation-wide; each step is described below.

### 3.1 Set-up

Step-1 should start by evidencing the issue(s) (collating data) and communicating the requirement for change. Step-1 also involves identifying stakeholders. This includes (but not limited to): health and performance practitioners (e.g., physical training instructors, strength and conditioning coaches, dietitians/nutritionists); caterers and logisticians; welfare/pastoral support officers; leaders from all levels; and—importantly—the end users (soldiers, sailors and aviators) of the system being developed. Senior leader buy-in and support from all stakeholders must be secured for success (Bullock et al., 2023). Important at the outset is to develop shared understanding of the WSA strategic intent and likely benefits to all levels of the organisation. Making these benefits relevant to different stakeholders will help instil belief and collective confidence to change.

Set-up also involves a Joint Strategic Needs Assessment (JSNA), where the defined health and performance environment/context—including existing (knowledge, skills) capabilities and (time resource) capacities, people (practitioners/leaders), physical (gyms, dining facilities) assets and (education, training) materials *currently* providing support to healthy behaviours and human performance—is mapped. The JSNA must identify critical gaps (in resources/assets, capabilities and capacities) that need addressing through the intervention(s). Thus, an evidenced picture of the WSA defined domain is systematically built. Facilitators and barriers supporting or impeding system delivery are identified; barriers are reviewed to determine if local management is possible, or if escalation up the chain of command is necessary to inform action or flag risk.

### 3.2 Consultation

Step-2 brings stakeholders together from across the system to capture information on causes of poor health and barriers to positive health behaviours and performance from *their* perspectives. This includes examining health and performance data (and data veracity) to provide empirical evidence for action, but should also collate stakeholders’ lived experiences. Consultation is vital for developing and agreeing on a shared WSA vision. Through engaging and involving the WSA pertinent parties (Fallowfield et al., 2024), the intent is to articulate acceptable and feasible health and performance improvement options to meet the needs and available resources (Step-1). This consultation also allows identification of challenges to implementation and discussion of solution options. Finally, strategic relationships should be purposefully developed between stakeholders and communication mechanisms agreed.

### 3.3 Planning

Step-3 must identify—and secure stakeholder agreement for—the WSA priorities (e.g., relative to health challenge and/or human performance requirements, solution feasibility and/or likely impact). The intervention(s) must be scoped and developed, and implementation planned. Options for development and implementation should be discussed, as should the implementation strategies and phases (i.e., iterative or “big bang”), timeframe (milestones) and resource commitment decisions. Step-3 also requires governance and assurance methods to be determined and agreed, ideally exploiting existing policies, procedures, capabilities, and ways of working where possible. This ensures efficiencies of resource use, and hence reduces likely barriers to implementation arising from new resource requirements. Stakeholders should also agree on the evaluation method and key performance indicators (KPI) for measuring effectiveness (i.e., what is *success* to the unit, its people, and how measured), as well as methods to capture lessons for continuous improvement.

### 3.4 Action

Step-4 involves WSA implementation and monitoring (i.e., action), ideally as a Quality Improvement Programme (QIP) to exploit evidence-based frameworks supporting well-structured evaluation (Glasgow et al., 2019; Pinnock et al., 2017). This should not be seen as “start-and-forget”; the system needs purposeful directing to be successful. Metric data should be collected that aligns with the requirements and priorities (Step-2), and data monitoring, analysis and evaluation initiated to allow feedback reporting (to individuals, practitioners, and commanders), acknowledging data protection regulations. An integrated, secure data ecosystem—providing timely feedback, raising individual awareness for action, informing data-enabled practitioner support, monitoring organisation health and performance risks—is a prerequisite for effective, person-centred, context-relevant WSA intervention(s). High-quality data are required to develop population-specific (MSKI and ill-health) risk stratification models and algorithms for personalising e-health technology intervention delivery solutions (Fallowfield et al., 2024c). This use of technology—tailored to military workforce and context requirements—could support resource-efficient, scalable programmes and interventions.

### 3.5 Manage

Step-5 involves maintaining and developing the system. Through Step-5, the organisation embeds Health in All Policies (HiAP) (Greer et al., 2022; World Health Organization, 2015) and system-thinking into structures, policies, processes, and programmes (education and training). This integration of the WSA intervention(s) into the fabric of the organisation is necessary for realising the paradigm shift to a new health and performance “business as usual” *System for Health* model.

### 3.6 Reflect and refresh

Finally, Step-6 concerns purposefully reflecting and refreshing the system; evaluating inputs and outputs to inform continuous improvement of the necessary organisational change management processes. New ways of working could be required, along with better alignment of existing practices, which should be informed by data. All stakeholders should critically review the HiAP-WSA model, considering opportunities for adapting to new requirements if required, improving service delivery, and strengthening the model to meet individual and organisational needs and wants. Indeed, system monitoring and/or organisational output and/or population outcome data should inform any modifications (e.g., changes in prioritisation) and improvements, maintaining an audit trail of development and implementation activities.

## 4 Measuring effect and return on investment from improving human performance

Measuring policy effect and return on investment are essential for realising enduring benefits from health and performance interventions. This provides objective feedback on whether desired outcomes are achieved, and can support programme justification and continuous improvement (Adams and Neville, 2020). Moreover, as interventions in military organisations are largely funded from public monies, these “checks and balances” are essential for accountability. The data ecosystem developed during Step-4 of operationalising a WSA, as well as supporting system delivery, will inform intervention effectiveness and cost-benefit calculations.

Measuring the effect of a WSA is challenging (Fallowfield and Carins, 2025). Indeed, determining “costs” *per se* of people-related policies in publicly funded, fiscally constrained defence organisations has been impossible (Haythornthwaite, 2023). Measurement should adopt a systems stance, rather than a simple linear view, given implementation is systemic (Renger et al., 2019). A systems stance recognises the complex interconnected nature of the problem, including the presence of factors beyond WSA implementation that could influence evaluation, as well as dynamic feedback loops (Renger et al., 2019). Additionally, measurement should move beyond short-term outcomes, to understand longer-term benefits to individuals, organisations, and society (Belcher and Palenberg, 2018). Furthermore, estimation of the return on investment from taking a WSA is desirable to demonstrate the value generated from implementation (Meacock, 2019).

**FIGURE 1:**
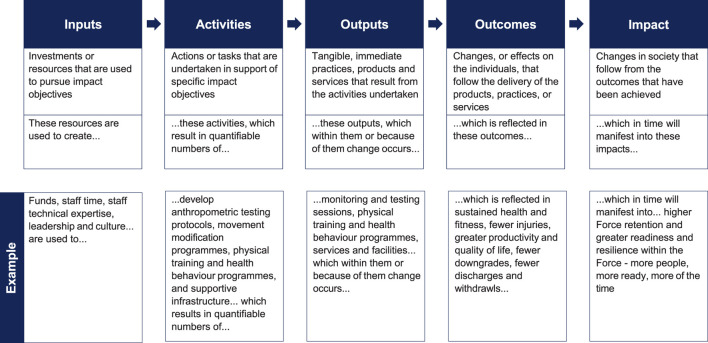
Impact value chain analysis for understanding the “value-adding” of a whole system approach to promoting health and human performance in military settings.

Measuring the effect of a WSA on health and human performance in a military setting can be achieved using Social Return on Investment (SRoI) analyses (Carins et al., 2024). This method benefits from a framework to guide evaluation, as well as a means of determining monetary returns. The SRoI process comprises several steps (Cabinet Office of the Third Sector, 2012): mapping the impact value chain to include inputs, activities, outputs, outcomes and impact (Figure 1); determining indicators for the monetary value of the desired outcomes; determining the likelihood of achieving those outcomes (from forecasting or evaluation); and calculating the combined value of all outcomes. A ratio is calculated to determine whether a positive return on investment is produced. This remains an evolving area of evaluation, but SRoI can assist in efficient and effective resource allocation (Maier et al., 2015). Measuring and valuing social impacts arising from outcomes—such as improved health and or performance—remains somewhat subjective compared with economic outcomes, necessitating a principled analytical approach. SRoI principles include: understanding sought or likely outcome changes, and how they are measured; valuing the things that matter; not over-claiming; and providing transparency regarding information used to establish judgments (Cabinet Office of the Third Sector, 2012).

Applying SRoI to evaluate a WSA to military health and human performance requires the WSA to be mapped to identify the relevant inputs, activities, outputs, and desired outcomes along with the likely longer-term impacts manifest from those outcomes. Metrics for these must be identified and methods for collecting metric data created. Indicators for the value of outcomes must be sourced to enable SRoI calculation. Health and human performance are widely considered *valuable* elements of Force readiness and military capability, but estimating the organisational value—and hence making the business case to resource programmes—has proved difficult. Thus, SRoI as a means of measuring effect and determining value can support prioritisation of investment for policy and programme change.

## 5 Policy courses of action

Employers have a responsibility to support a fit Force to meet *military readiness* requirements (Fallowfield and Carins, 2025). However, workforce shortages and increasing operational demands hinder leaders’ abilities to prioritise health and fitness. Table 1 outlines three courses of action, their implications, and challenges. Without change, problems may worsen. Non-systemic actions may offer some benefit. However, despite acknowledged delivery challenges, the WSA has greater potential for impactful and enduring health and performance improvements.

**TABLE 1:** Policy courses of action for supporting health and fitness, potential implications/outcomes and challenges for policy delivery.

Course of action	Implications - Outcomes	Challenges to delivery
No change	High MSKI/ill-health prevalence persist Further specialisation of roles add to MSKI/ill-health prevalence Workforce capacity and capability continue to decrease Increasing demands placed on each Service person, especially operationally facing roles Individual and Team performances, at best, do not improve; at worst, further decline	No challenges; organisation continues business as usual
Non-systemic approach	Likely to produce some immediate changes or localised areas of positive change Reduced effectiveness of single strategies due to lack of purposeful coherence Inefficiencies in resource allocation through uncoordinated activities Risk of conflicting policies and actions (gains made through one action defeated by inaction or contradictory action elsewhere) Likely effects diminish over time due to military posting cycles (“people churn”); action is lost	Leadership prioritisation required at localised or small-scale level Stakeholder engagement required with single or separate strategies Ensuring knowledge/understanding of benefits Securing resource requirement Training requirements for policy delivery Implementation and evaluation require multiple resources and potentially duplication of effort
Whole system approach	Works upstream to promote good health and mitigate ill-health; reduced financial cost of people capability Looks for synergies—and hence efficiencies—between health/fitness and other core people objectives Fosters collaborative working; develops shared understanding and organisational cohesion Avoids causing harm; mitigates unintended consequences *a priori* Should be more effective—and hence financially efficient—in generating and maintaining a people capability Action over multiple levels and locations encourages cultural change and enduring *real* organisational change (less likely to be disrupted by change in personnel)	Leadership prioritisation required at multiple locations and levels Multiple stakeholder engagement across the WSA actions Ensuring knowledge/understanding of benefits Resource requirements, but securing resource may be easier as multiple beneficiaries to outcomes Training requirements for policy delivery Developing cross-function understanding and working practices requires organisational change Implementation and evaluation require broader resources, following aligned protocols, but is likely to include complexity due to size and scale

## 6 Conclusion

Military health provides the foundation for human performance, enhancement and/or augmentation, realising a more present, more capable and more resilient Force. We propose a WSA to set the organisation conditions that—*vitally* in hierarchical, military organisations—enable individuals and teams to thrive as a healthy, fit Force. Ill-health and MSKI risk degrade the deployable people capability, whereas a unified *System for Health* would better support this capability. Our six-step model develops an embedded, integrated, context-specific, person-centred WSA for health and human performance, fostering widespread stakeholder buy-in and establishing, *a priori,* effective mechanisms for planning, delivery, evaluation and continuous improvement. Organisations tend to be constrained by the perceived barriers to intervention implementation rather than focussing on realisable opportunities from change. Measuring benefits and return on investment can bring transparency to this risk-balance judgement. *Valuing* good health and human performance—providing benefits in monetary terms—should realise better support to Force readiness and enhance the “people component” of operational effectiveness.
